# PDCD10-Deficiency Promotes Malignant Behaviors and Tumor Growth via Triggering EphB4 Kinase Activity in Glioblastoma

**DOI:** 10.3389/fonc.2020.01377

**Published:** 2020-08-07

**Authors:** Xueyan Wan, Dino Vitali Saban, Su Na Kim, Yinlun Weng, Philipp Dammann, Kathy Keyvani, Ulrich Sure, Yuan Zhu

**Affiliations:** ^1^Department of Neurosurgery, University Hospital Essen, University of Duisburg-Essen, Essen, Germany; ^2^Department of Neurosurgery, Tongji Hospital, Tongji Medical College, Huazhong University of Science and Technology, Wuhan, China; ^3^Institute of Neuropathology, University Hospital Essen, University of Duisburg-Essen, Essen, Germany

**Keywords:** EphB4 and EphB4 kinase inhibition, PDCD10/CCM3, lentivirus mediated shRNA transduction, glioblastoma, tumor malignant behavior

## Abstract

We previously reported an angiogenic and tumor-suppressor-like function of programmed cell death 10 (PDCD10) in glioblastoma (GBM). However, the underlying mechanism remains to be elucidated. We hypothesized that loss of PDCD10 activates GBM cells and tumor progression via EphB4. To this end, PDCD10 was knocked down in U87 and T98g by lentiviral mediated shRNA transduction (shPDCD10). GBM cell phenotype *in vitro* and tumor growth in a mouse xenograft model were investigated in presence or absence of the treatment with a specific EphB4 kinase inhibitor NVP-BHG712 (NVP). We demonstrated that knockdown of PDCD10 in GBM cells significantly upregulated the mRNA and protein expression of EphB4 accompanied by the activation of Erk1/2. EphB4 kinase activity, reflected by phospho-EphB4, significantly increased in shPDCD10 GBM cells, and in tumors derived from shPDCD10 GBM xenografts, which was abolished by the treatment with NVP. Furthermore, NVP treatment significantly suppressed PDCD10-knockdown mediated aggressive GBM cell phenotype *in vitro* and extensive tumor cell proliferation, the tumor neo-angiogenesis, and a quick progression of tumor formation *in vivo*. In summary, loss of PDCD10 activates GBM cells and promotes tumor growth via triggering EphB4. Targeting EphB4 might be an effective strategy particularly for the personalized therapy in GBM patients with PDCD10-deficiency.

## Introduction

Glioblastoma (GBM) is the most aggressive and common primary brain tumors in adults. The etiology and pathogenesis of GBM remains unclear. The median patient survival is <15 months and the 5-year survival rate is <5% despite extensive therapy including surgical resection, radiotherapy, and chemotherapy (1–3). Neo-angiogenesis is a specific characteristic of GBM, which distinguishes GBM from low-grade gliomas and is associated with a high risk of tumor recurrence, invasive growth, and multi-therapy resistance. Thus, anti-angiogenesis has become promising strategy for GBM treatment (2, 4). However, the currently available anti-angiogenic drugs cannot ultimately improve the survival of GBM patients (5), and moreover, there is no drug available specifically targeting tumor invasion. Therefore, the development of new therapeutic targets for GBM could succeed against this capacity.

The programmed cell death 10 (PDCD10) gene encodes an evolutionarily conserved protein and is ubiquitously expressed in nearly all human tissues and various types of cells (6). PDCD10 was initially named TF-1 cell apoptosis-related gene 15 (TFAR15) because of its upregulation upon apoptotic stimuli. The apoptotic function of PDCD10 appears cell type-dependent (7, 8). PDCD10 is also known as cerebral cavernous malformation 3 (CCM3). A loss-of-function mutation in PDCD10 causes the familial form of cerebral cavernous malformation, one of the most common vascular lesions in the central nervous system involving aberrant angiogenesis (9, 10). We (8, 11, 12) and others (13, 14) have demonstrated the crucial roles of PDCD10 in endothelial proliferation, apoptosis, senescence, and autophagy. The underlying signaling pathways including EphB4-Notch-p-Erk, VEGFR2, STK24/25, MST4, RhoA, Smad, MEKK3-KLF2/4, and mTOR have been identified. However, the fundamental roles of this protein in tumors remain less understood. Nevertheless, increasing evidence has demonstrated the involvement of PDCD10 in different types of tumors. Patients harboring heterozygous mutations of PDCD10 displayed a high risk of developing meningioma (15–17). Downregulation of PDCD10 was associated with chemo-resistance in colorectal cancer cells (18). Another study reported that microRNA-103 suppressed tumor cell proliferation by targeting PDCD10 in prostate cancer (19). We demonstrated that downregulation of PDCD10 in human primary GBM was associated with the activation of Akt, higher tumor microvessel density, and higher grade of peritumoral edema. More interestingly, we observed the absence of PDCD10 immunoreactivity in the majority of endothelia of tumor vessels and in tumor cells (20). These data, for the first time, implicated PDCD10 in human GBM.

Ephrin type-B receptor 4 (EphB4), a member of tyrosine kinase family, is preferentially expressed in venous endothelial cells (ECs). EphB4 binds specifically with its ligand ephrinB2 via direct cell-cell contact, resulting in EphB4-ephrinB2-complex dimerization, and a subsequent induction of bi-directional signaling. Upon engagement of EphB4 with ephrinB2, EphB4 becomes auto-phosphorylated on its kinase domain, thereby activating the kinase-dependent forward signaling; the reverse signaling is activated upon ephrinB2 tyrosine phosphorylation through recruitment of itself (21). This ligand-receptor interaction plays important roles in diverse cell biological processes, such as cell morphogenesis (22), cell adhesion/repulsion (23), and angiogenesis (24), and is also involved in tumor progression (25). EphB4 is overexpressed in various type of solid tumors, such as colon cancer (26), bladder cancer (27), breast cancer (28), esophageal cancer (29), lung cancer (30), and mesothelioma (31). It is noteworthy that EphB4 expression increased in human gliomas in a grade-dependent manner and was also associated with the neo-vascularization and with tumor progression and poor prognosis in GBM patients (32, 33). These data suggest EphB4 as a critical intermediator in GBM.

We have previously investigated the angiogenic and apoptotic functions of PDCD10 in vascular endothelial cells (ECs) and the underlying molecular mechanism (8, 11, 12). By siRNA transfection- and lentiviral shRNA transduction mediated knockdown methods, we demonstrated that loss of endothelial PDCD10 stimulated endothelial proliferation, adhesion, migration, and tube formation *in vitro* and promoted neo-angiogenesis *in vivo* through the activation of EphB4-forward signaling (12). Further study of the interaction between ECs and GBM cells (TCs) indicated that PDCD10-ablation in ECs activated proliferation-, adhesion-, invasion-, and migration of TCs *in vitro* and promoted neo-angiogenesis and tumor growth in xenograft models (34). These data provided evidence for the crucial impact of EC-originated PDCD10 in GBM progression. Based on these findings together with the previous observation that PDCD10 was absent not only in the majority of ECs but also in TCs of the tumor tissue resected from GBM patients (20), we recently extended our research to study the role of TC-originated PDCD10 in GBM. As expected, upon knockdown of PDCD10 TCs activated themselves toward a more aggressive phenotype and promoted tumor growth (35). However, its underlying mechanism remains unclear. In this study, we speculate that EphB4 is the target of PDCD10 in GBM cells and that interfering EphB4 signaling could rescue the phenotype of PDCD10-deficient GBM cells and suppress tumor growth in a mouse model of GBM.

## Materials and Methods

### Culture of Lentiviral-Mediated shRNA Transduced GBM Cells and Cell Treatment

The establishment of the stable knockdown of PDCD10 (shPDCD10) and controls (empty vectors, EV) in two human GBM cell lines, U87 and T98g, by using two different lentiviral mediated shRNA transduction systems has already been previously described (35). The transduced U87 cells were cultured in DMEM containing doxycycline (dox, 1 μg/ml) (Sigma-Aldrich, Munich, Germany) to maintain the knockdown of PDCD10 and puromycin (1 μg/ml) (Sigma-Aldrich, Munich, Germany) to eliminate non-transduced cells. The transduced T98g cells were cultured in MEME containing only puromycin (1 μg/ml) (Sigma-Aldrich, Munich, Germany) because of the permanent knockdown status of PDCD10. The successfully transduced U87 cells produced red fluorescent protein (RFP) whereas the T98G cells expressed green fluorescent protein (GFP) leading to red and green fluorescence, respectively.

To inhibit EphB4 kinase activity, NVP-BHG712 (NVP), a specific EphB4 kinase inhibitor, was purchased from Sigma (Sigma-Aldrich, Munich, Germany), and dissolved in DMSO as stock solution. Cells were treated with a solution containing 10 or 25 nM of NVP for different period of time as indicated for each experiment.

### Real-Time RT-PCR (RT^2^-PCR)

Total RNA was extracted using the innuPREP RNA mini kit (Analytik jena, Berlin, Germany). cDNA was synthesized using the iscript cDNA kit (Bio-Rad, Munich, Germany). The PCR reaction mixture was prepared to a final volume of 15 μl comprising of 6 μl of cDNA template (4 ng/μl), 7.5 μl of SYBR green supermix (Bio-Rad, Munich, Germany), 0.3 μl of forward and reverse specific primers (10 μM), and 0.9 μl RNase-free H_2_O. Real-time PCR was performed according to our recent publication. Glyceraldehyde-3-phosphate dehydrogenase (GAPDH) was stably detected and was used as the housekeeping gene. The relative expression of target gene was calculated by 2^−ΔΔCt^ method as described previously (34). Primer sequences and annealing temperature for individual genes are listed in Table 1.

**Table 1:** Primer sequences for real-time RT-PCR.

**Primer**	**Sequence**	**Ta (^**°**^C)**
*PDCD10*		60
for.	TGG CAG CTG ATG ATG TAG AAG	
rev.	TCG TGC CTT TTC GTT TAG GT	
*EphB4*		58
for.	TGT GTT GGA GGG AAC CTG TTT C	
rev.	GGG CCC CTG TTT CAA CTT G	
*GAPDH*		60
for.	AGC CAC ATC GCT CAG ACA	
rev.	GCC CAA TAC GAC CAA ATC C	

*for., forward; rev., reverse. Ta. Annealing temperature*.

### Cell Phenotype Study

Cells were first cultured in serum-free media for 24 h before phenotype study in order to synchronize cell cycle thereby minimizing the influence of proliferation on cell migration and invasion. Thereafter, the phenotype of U87 and T98g cells including migration, adhesion, and invasion was assessed according to our previous protocols (35). Briefly, for spheroid migration assay, U87 and T98g spheroid was formed after overnight incubation with 20% of methylcellulose solution. The spheroids were reseeded into the plate pre-coated with matrigel. The spheroid diameter was measured at different time points by Image J software. For transwell invasion assay, GBM cells were suspended in serum-free medium and seeded into the insert precoated with matrigel. The medium containing 10% of FBS was added into the lower chamber. After incubation for 24 h, the uninvaded cells were gently removed with a cotton swab. The invaded cells were stained with crystal-violet (0.5%) and destained with sodium dodecylsulfate (1%) followed by measuring the absorbance at 550 nm by a plate reader. For extracellular matrix (ECM) adhesion assay, cells were seeded in 96-well-plate pre-coated with matrigel and incubated for 90 min. After gently removing the non-adherent cells, the adherent cells were detected after crystal-violet staining and the absorbance was measured at 550 nm by a plate reader. For cell growth assay, 2,000 cells were seeded on the 96-well-plate and the cells were treated with a DMSO solution containing 10 nM of NVP or the only DMSO. The cell population was measured through the intensity of the fluorescence on plate reader (TECAN, Maennedorf, Switzerland) at 24, 48, and 72 h after the treatment. For T98g transduced cells (GFP), the excitation wavelength was 485 nm and the emission wavelength was 535 nm; for U87 transduced cells (RFP), the excitation wavelength was 553 nm and the emission wavelength was 574 nm.

### Western Blot

Total protein extraction and Western blot were performed as described previously (12, 35). The following antibodies were used: PDCD10 (1:400; Atlas Antibodies); EphB4 (1:400, Santa Cruz), p-Erk1/2, p-Akt, and GAPDH (each 1:1000 dilution, Cell Signaling Technology).

### Enzyme-Linked Immunosorbent Assay (ELISA)

After treatment with NVP (10 nM) for 90 min, cell pellets were collected and proteins were extracted. The level of phosphor-EphB4 (p-EphB4), reflecting the kinase activity of EphB4, was measured according to the manufacture's instruction by using an ELISA kit (R&D, Wiesbaden, Germany).

### Glioblastoma Xenograft Model and the Treatment

Ethic approval was obtained (No. 84-02.04.2012.A348) and the animal experiments were performed accordingly. The glioblastoma cell line derived xenograft model was performed in female nude mice (4–6 weeks old) as previously described (34, 35). Briefly, to maintain the knockdown of PDCD10, transduced U87 cells were cultured in the complete medium with doxycycline (1 μg/ml) (shPDCD10), whereas the transduced U87 cells cultured without doxycycline (control U87) served as control (*n* = 8 for each group). Cell suspension (100 μl) was prepared with 1 million of shPDCD10-U87 or control-U87 cells. Then the individual cell suspension was mixed with VEGF (1,000 ng/ml) (R&D Systems, Wiesbaden-Nordenstadt, Germany), bFGF (1,000 ng/ml) (R&D Systems) and 100 μl of Matrigel (BD, Heidelberg, Germany). The mixture was subcutaneously implanted into the left flank of the nude mice. To maintain the stable knockdown of PDCD10 *in vivo*, doxycycline (2 mg/ml), and 1% sucrose (Sigma, München, Germany) was added to their drinking water. To investigate whether inhibition of EphB4 could rescue shPDCD10-induced tumor growth, the mice implanted with shPDCD10-U87 were treated with NVP (8 mg/kg, i.g.) every 2 days beginning on the sixth day after implantation. The control mice received the same amount of solvent without treatment [the solvent containing 10% v/v 1-methyl-2-pyrrolidone (Sigma, Munich, Germany) and 90% v/v polyethylene glycol 300 (Sigma, Munich, Germany)]. Tumor size was monitored twice a week by caliper. Tumor volume (TV) was calculated according to previous description (35). Mice were euthanized and tumors were excised 3 weeks after the implantation. Tumor mass was weighed and divided into three pieces for RT^2^-PCR, Western blot, ELISA, and sectioning and staining.

### Immunohistochemistry Staining

Immunohistochemistry was performed according to the previously described protocol (35). Following antibodies were used: mouse anti-Ki-67 (1:200, Zytomed, Berlin, Germany) and mouse anti-CD31 (1:40, Dako, Hamburg, Germany). Images were acquired using a microscope (Axio Imager M2, Zeiss, Oberkochen, Germany).

### Database Analysis and Statistical Analysis

Using the TCGA-GBM dataset obtained by RNA sequencing, transcriptional levels of PDCD10, and EphB4 were preliminarily analyzed by a free web tool GlioVis (http://gliovis.bioinfo.cnio.es/). Based on expression data, a correlation coefficient (*r*) between their expressions in cohort was calculated at the 0.01 level (2-tailed).

Data are presented as mean ± SD. Statistical analysis was performed using the WinSTAT and Graphpad Prism 8 software. Results among groups were analyzed by the analysis of variance (36). A *P* < 0.05 was considered statistically significant.

## Results

### Up-Regulation of EphB4 Expression and Activation of EphB4 Kinase Activity in PDCD10-Knockdown GBM Cells

Next generation sequencing revealed that transcription levels of EphB4 in GBM were drastically increased than those in non-tumor (data not shown; log2FC = 2.33; *P* < 0.0001). In order to study if such a significant increase in EphB4 expression levels in GBM is related to PDCD10 expression, we first explored the TCGA-GBM dataset. In cohort of 156 patients, a weak negative correlation was found [Supplementary Figure 1; *r* = −0.206; significant level = 0.010 (2-tailed)].

**Figure 1:**
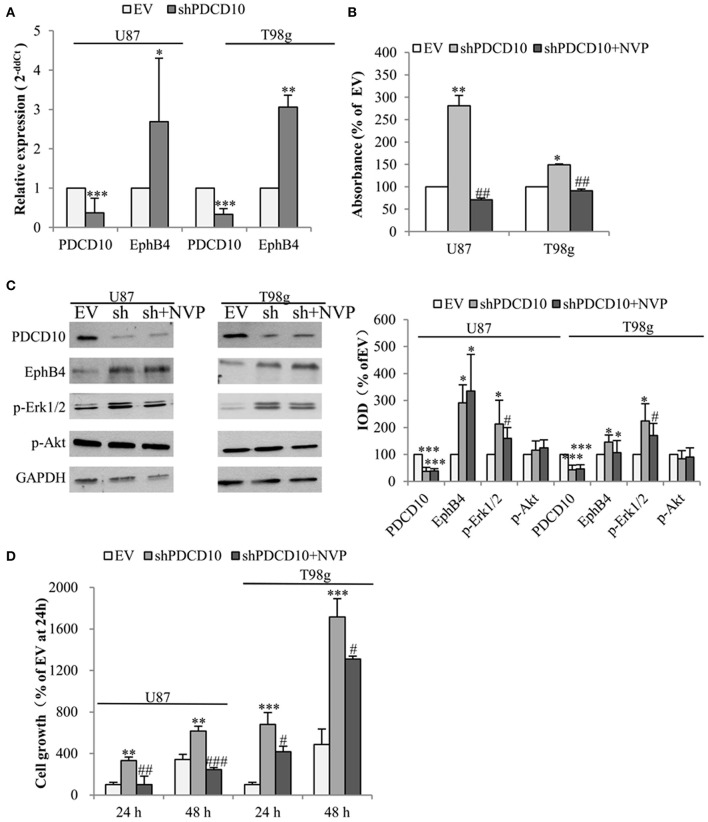
Knockdown of PDCD10 increases the expression and the kinase activity of EphB4 in GBM cells and promotes cell growth, which is reversed by the treatment with the specific inhibitor of EphB4 kinase NVP. PDCD10 was knocked down in U87 or T98g cells by lentiviral shRNA transduction. **(A)** RT^2^-PCR demonstrated a significant downregulation of PDCD10 mRNA and an upregulation of EphB4 mRNA in PDCD10 knockdown (shPDCD10) U87 and T98g cells in comparison to EV-transduced cells. **(B)** ELISA demonstrated that knockdown of PDCD10 elevated the level of p-EphB4, reflecting the activity of EphB4, which was reversed upon the treatment with NVP (10 nM), a specific EphB4 kinase inhibitor. **(C)** Western blot revealed that knockdown of PDCD10 increased the EphB4 protein expression and activated Erk1/2 but not Akt in GBM cells (left). The activation of Erk1/2 mediated by PDCD10 knockdown was partially reversed by the treatment of NVP. Semiquantification of the blots was performed measuring the integrated optical density (IOD) of the blots normalized to the IOD of housekeeping protein GAPDH (right). **(D)** Knockdown of PDCD10 accelerated GBM cell growth. This effect was reversed by the treatment with NVP for 24 or 48 h as shown by fluorescence measurement of U87-(RFP) and T98g-(GFP) cells. For transduced U87 cells expressing RFP, the excitation wavelength was 553 nm and the emission wavelength was 574 nm. For transduced T98g cells expressing GFP, the excitation wavelength was 485 nm and the emission wavelength was 535 nm. All data presented in **(A–D)** were representative of at least three independent experiments. *P < 0.05, **P < 0.01, and ***P < 0.001, compared with EV; ^#^*P* < 0.05, ^*##*^*P* < 0.01, ^*###*^*P* < 0.001, compared with shPDCD10.

To check the influence of PDCD10 on EphB4 expression in GBM cells, the level of EphB4 mRNA, protein, and p-EphB4 were studied in two different GBM cell lines U87 and T98g in which PDCD10 were stably knocked down by shRNA transduction. As detected by RT^2^-PCR, the expression of PDCD10 in shPDCD10-U87 cells and shPDCD10-T98g cells was reduced to 37 and 33% of the control, respectively (both *P* < 0.001), concomitantly accompanied by a 2.7-fold (*P* < 0.05) and a 3.1-fold (*P* < 0.01) up-regulation of EphB4 mRNA (Figure 1A). ELISA for p-EphB4, reflecting the kinase activity of EphB4, showed a 280% (*P* < 0.01) and 150 % (*P* < 0.05) increase in the level of p-EphB4 in shPDCD10-U87 and shPDCD10-T98g cells in comparison to the controls, respectively (Figure 1B). Furthermore, PDCD10 knockdown-mediated activation of EphB4 was abolished by the specific EphB4 kinase inhibitor NVP in both U87- and T98g cells. Western blot confirmed a significant downregulation of PDCD10 protein expression in shPDCD10-U87- and shPDCD10-T98G cells (both *P* < 0.001), accompanied by an activation of Erk1/2 but not Akt (Figure 1C; left panel). Semiquantitative analysis showed a 2.9-fold (*P* < 0.05) and a 2.1-fold (*P* < 0.05) up-regulation of EphB4 and p-Erk1/2 in shPDCD10-U87 cells, respectively; a 1.5-fold (*P* < 0.05) and a 2.2-fold (*P* < 0.05) up-regulation of EphB4 and p-Erk1/2 in shPDCD10-T98g cells were detected (Figure 1C; right panel). NVP treatment partially reversed the activation of Erk1/2 derived by PDCD10 knockdown. Upregulated EphB4 protein level in shPDCD10 was not influenced by NVP (Figure 1C), which is however not surprising as NVP selectively inhibits autophosphorylation of EphB4, but does not influence the EphB4 gene transcription or protein translation processes. Furthermore, we examined the effect of NVP on GBM cell growth. Knockdown of PDCD10 (shPDCD10) stimulated U87 (*P* < 0.01) and T98g (*P* < 0.001) cell growth, which was significantly inhibited by the treatment with NVP (both *P* < 0.05) (Figure 1D).

### Inhibition of EphB4 Kinase Activity Rescues the Phenotype of shPDCD10-GBM Cells *in vitro*

**Figure 2:**
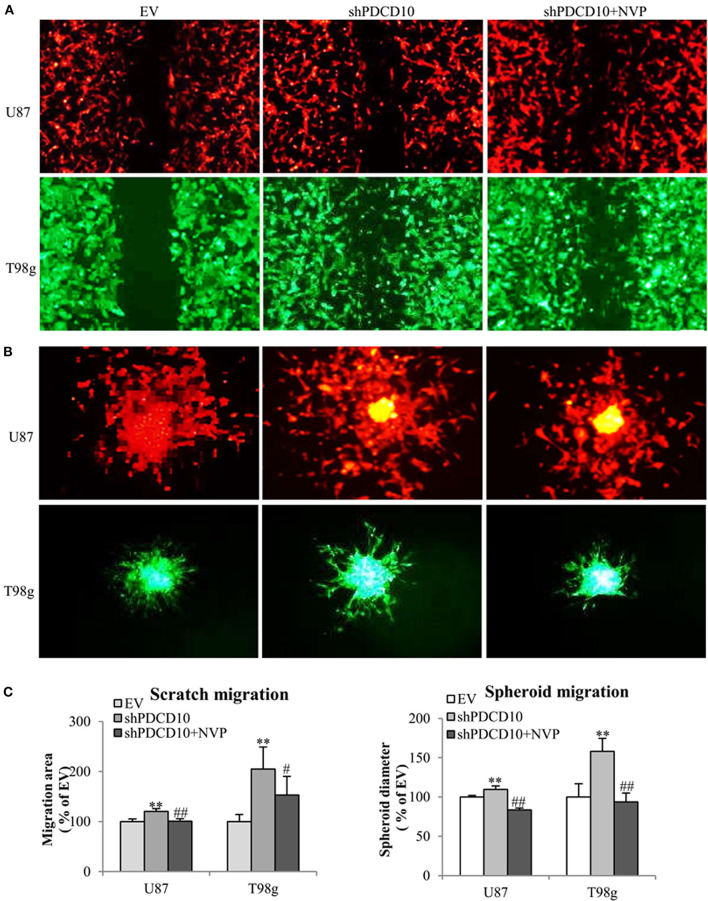
Treatment with NVP attenuates PDCD10 knockdown-induced migration of GBM cells. shPDCD10-GBM cells were treated with NVP (10 nM) for different time periods as indicated in individual experiments. EV served as control. **(A)** Scratch assay: cells were treated with 10 nM of NVP for 24 h followed by the scratching. The migration was recorded 24 h after scratching. **(B)** Spheroid migration assay: the spheroids formed by GBM cells were suspended in normal medium and seeded on 96-well-plates precoated with matrigel. After treatment with NVP (25 nM) for 48 h, the spheroids were collected and their diameter was measured. Scale bar: 100 μm. **(C)** Statistical analysis of scratch assay and spheroid migration assay. **P < 0.01, compared with EV; ^#^*P* < 0.05, ^*##*^*P* < 0.01, compared with shPDCD10.

In order to investigate effects of EphB4 activity on the behavior of GBM cells upon PDCD10 knockdown, migration-, adhesion-, and invasion of shPDCD10-U87- and shPDCD10-T98g cells were evaluated with and without NVP treatment. As demonstrated by scratch assay (Figure 2A) and spheroid assay (Figure 2B), PDCD10 knockdown resulted in a remarkable increase in U87 and T98g migration, which was reversed by the treatment with NVP. The quantitative analysis of the migration of both types of cells and in two migration assay methods were shown in Figure 2C.

**Figure 3:**
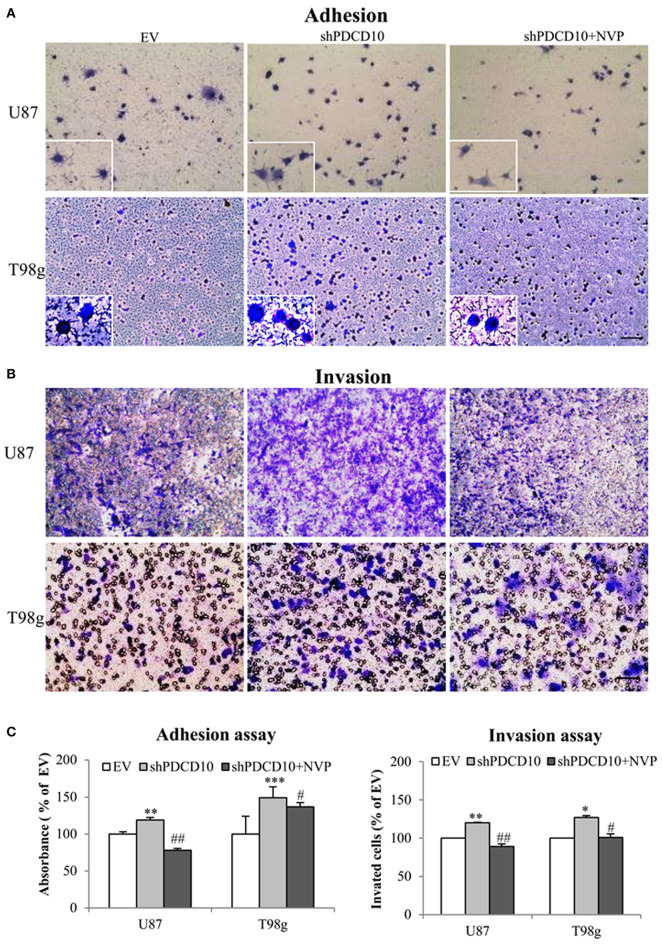
Treatment with NVP inhibits PDCD10 knockdown-induced adhesion and invasion of GBM cells. The adherent U87- or T98g cells were detected by crystal-violet staining after treatment of NVP (25 nM, 90 min) following by the absorbance measurement at 550 nm **(A,C)**. The invasion of U87- or T98g cells were evaluated by the trans-well assay after treatment of NVP (25 nM for 48 h), the invaded cells were stained by crystal-violet and the absorbance was measuring at 550 nm **(B,C)**. All data presented were representative of at least three independent experiments. *P < 0.05, **P < 0.01, ***P < 0.001 compared with EV; ^#^*P* < 0.05, ^*##*^*P* < 0.01, compared with shPDCD10. Scale bar: 100 μm.

Knockdown of PDCD10 also promoted adhesion (Figure 3A) as well as invasion (Figure 3B) of U87 and T98g GBM cells compared to the corresponding EV controls. Of note, the treatment with NVP significantly inhibited these activated GBM cell phenotypes resulted from PDCD10 knockdown (Figure 3).

### Inhibition of EphB4 Kinase Activity Suppresses Tumor Progression *in vivo*

**Figure 4:**
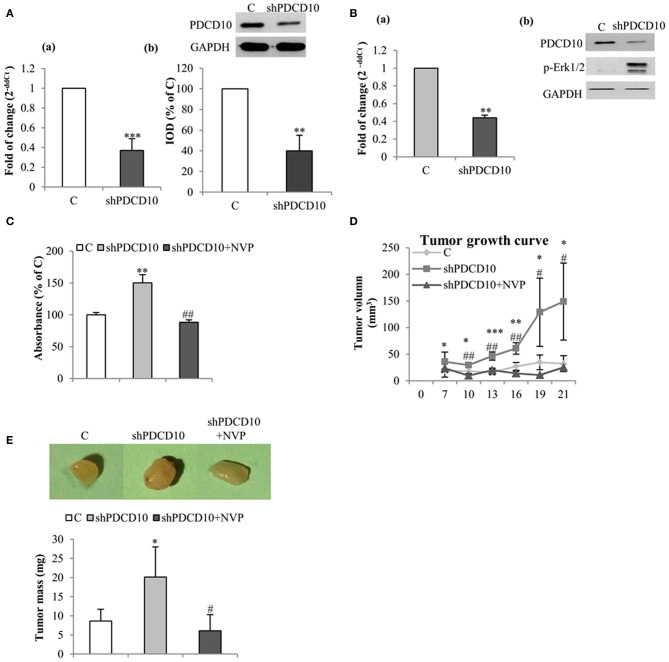
Inhibition of EphB4 suppresses PDCD10-knockdown mediated EphB4 activation and the tumor growth in a human GBM xenograft model in mice. **(A)** Confirmation of the knockdown of PDCD10 mRNA (a) and protein (b) in U87 cells before the implantation. **(B)** Expression of PDCD10 in resected tumors at mRNA (a) and protein levels (b) determined by RT^2^-PCR and Western blot, respectively. **(C)** ELISA for p-EphB4 demonstrated the activation of EphB4 in PDCD10-knowckdown tumors, which was completely reversed after treatment with NVP. **(D)** The tumor growth curve revealed a significantly faster tumor progression in PDCD10-knockdown tumors and a marked tumor growth inhibition by the treatment with NVP. **(E)** NVP treatment reduced significantly the tumor mass compared to that from PDCD10-knowckdonw mice. Upper: the representative images show the tumors from control, shPDCD10, and NVP-treated shPDCD10-mice, respectively. Lower: statistical analysis, *P < 0.05, **P < 0.01, ***P < 0.001, compare to control; ^#^*P* < 0.05, ^*##*^*P* < 0.01, compare to shPDCD10 (*n* = 7, each group).

To further investigate the role of EphB4 in tumor formation and growth, shPDCD10-U87 cells were subcutaneously implanted to nude mice. Before the implantation, the knockdown of PDCD10 was confirmed at mRNA- (Figure 4Aa) and protein levels (Figure 4Ab; corresponding blots in upper panel) in GBM cells. Three weeks after the implantation, tumors were resected and weighed before being analyzed by RT^2^-PCR, Western blot, ELISA, and tissue staining. RT^2^-PCR (Figure 4Ba) and Western blot (Figure 4Bb) confirmed a stable knockdown of PDCD10 in tumors derived from transplanted shPDCD10-U87 cells. Part of the protein lysate (produced for Western blot) was used to perform ELISA for p-EphB4. We found a drastically increased level of p-EphB4 in the PDCD10-knockdown tumors compared to the controls (Figure 4C). The NVP treatment completely reversed the upregulation of p-EphB4 in xenograft tumors of the PDCD10-knockdown mice Consequently, the tumor progression was significantly faster in the shPDCD10-mice than that in the controls as inspected from day 7 to day 21 after implantation (Figure 4D). The tumor mass weighted at the end of the experiment (21 days after implantation) was 2.3-fold in the shPDCD10-mice than that in the controls (*P* < 0.05) (Figure 4E). More importantly, the treatment with NVP not only abolished PDCD10-knockdown-mediated activation of EphB4 (Figure 4C), but also suppressed the aggressive tumor progression and rapid growth in shPDCD10 mice (Figures 4D,E).

**Figure 5:**
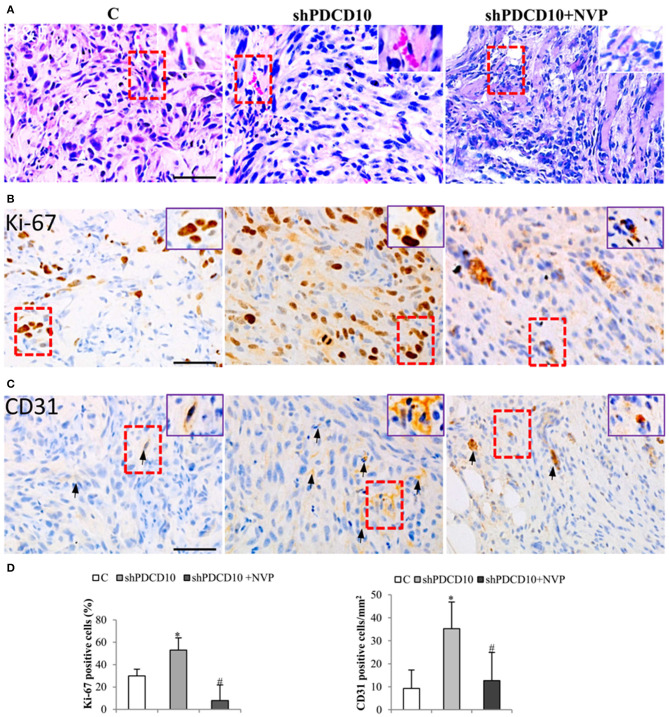
Knockdown of PDCD10 facilitates tumor cell proliferation and stimulates angiogenesis in a human GBM xenograft model in mice. **(A)** H&E staining revealed the histological feature of the GBM xenograft in all three groups. The vessel-like structure containing blood cells was observed in the tumors (arrows). A representative area of interest is given in inset at a higher magnification. **(B,C)** Immunohistochemistry with Ki67 **(B)** and CD31 **(C)** antibodies. **(D)** Statistical analysis of immunohistochemistry. Ki67 positive cells and CD31-positive cells were more frequently detected in shPDCD10-mice compared with the control and with NVP-treated shPDCD10 mice. *P < 0.05, compare to control; ^#^*P* < 0.05, compare to shPDCD10 (*n* = 7, each group).

Next, we performed histological staining and immunostaining on the resected xenograft tumors. H&E staining revealed cellular atypia, areas resembling necrosis with tumor cell palisading, and angiogenesis. Interestingly, microvessel-like structures with blood cells were observed in H&E stained sections (Figure 5A, arrows), suggesting a functional vascular network in xenograft tumors. Immunostaining for cell proliferation marker Ki67 revealed extensive Ki67-positive cells on the section from shPDCD10-mice (Figure 5B). Furthermore, immunostaining confirmed some scatted CD31-positive cells on the tumor sections (Figure 5C, arrows). Quantitative analysis demonstrated that that NVP treatment completely reversed the increased cell proliferation (*P* < 0.05, Figure 5D) and neo-angiogenesis (*P* < 0.05, Figure 5D) caused by PDCD10 knockdown.

## Discussion

PDCD10 is ubiquitously expressed and plays vital roles in vasculogenesis, angiogenesis, apoptosis, and autophagy. The biological and pathological functions of PDCD10 have been intensively studied in vascular endothelial system throughout the past decade (8, 11). Loss of endothelial PDCD10 affects vascular development and causes cerebral cavernous malformation (9, 10). We previously demonstrated a pronounced pro-angiogenic and anti-apoptotic effects in PDCD10 deficient endothelial cells, and identified a pivotal signaling pathway Dll4/Notch-EphB4-Erk1/2 underlying the endothelial functions of PDCD10 (11). More recently, the role of PDCD10 in malignant tumors has been emphasized. However, little is known about its role in GBM. Massive neo-angiogenesis is one of the distinct features of GBM, which distinguish GBM from other low-grade gliomas, and is a critical and causative factor leading to invasion, growth, and therapy-resistance seen in this tumor entity. In our previous study, we could demonstrate that PDCD10 was often absent in endothelial cells (ECs) of tumor vessels as well as in GBM cells (TCs) of human GBM; PDCD10 expression was associated with a higher microvessel density in GBM. Moreover, knockdown of either EC-originated or TC-originated PDCD10 activated GBM cells and promoted tumor growth via stimulating the release of a group of growth factors and angiogenic factors (34, 35). These data strongly support a crucial tumor suppressor-like function of PDCD10 in GBM, which raised our interest to further explore the mechanism underlying tumor-promoting effect mediated by shPDCD10 in GBM. The present study demonstrated that knockdown of PDCD10 in GBM cells led to a significant upregulation of EphB4 mRNA and protein expression and consequently a remarkable increase in the EphB4 kinase activity, which was completely reversed by the treatment with a specific EphB4 kinase inhibitor NVP. These results indicate that EphB4 acts downstream of PDCD10 in GBM cells. The functional study demonstrated that upon NVP treatment, the inhibition of EphB4 kinase activity can inhibit the aggressive behavior in shPDCD10 GBM cells, as well as hinder rapid tumor formation and proliferation, thereby leading to a reduction of the tumor mass in PDCD10 knockdown tumor. These findings provide evidence that the tumor-promoting effect resulted from PDCD10 knockdown is mediated by upregulation/activation of EphB4. Both forward and reverse signaling by the EphB4/ephrinB2 interaction is context-dependent and can vary from one cancer type to another (37). EphB4 is upregulated in GBM and EphB4/ephrinB2 pathway is involved in the neo-angiogenesis (38), tumors progression, prognosis of GBM (32, 33, 39), and in the resistance to anti-angiogenesis therapy (39). These data underscore the oncogenic function of EphB4 in GBM, and suggest EphB4 as a promising therapeutic target (40). Pharmacologically targeting EphB4 has emerged as a strategy against numerous cancers (41). Soluble EphB4 (sEphB4) is a soluble decoy of EphB4 that blocks EphB4-Ephrin-B2 bi-directional signaling. The anti-tumor activities of sEphB4 have been shown in multiple tumor models (42, 43). Another interesting option to inhibit EphB4 is the use of humanized monoclonal anti-EphB4. Krasnoperov et al. (44) described two EphB4 antibodies MAb131 and MAb47 that inhibit angiogenesis and tumor growth in a non–small-cell lung cancer cell line with distinct mechanisms. Of particular note, application of small molecule compounds that specifically act as a kinase inhibitor is more feasible to interfere with the activity of kinases such as EphB4. NVP is a small molecule that selectively inhibits tyrosine kinase activity of EphB4 (45). Becerikli et al. (46) showed that NVP inhibited EphB4 autophosphorylation, reduced cell growth rate of synovial sarcoma, and fibrosarcoma cells *in vitro* and hampered sarcoma lung metastasis *in vivo*. Inhibition of EphB4 by NVP overcomes the acquired resistance to cisplatin in melanoma xenograft models (47). By using NVP, we previously identified the EphB4-Erk1/2 as downstream signaling of Dll4-Notch pathway in endothelial cells. This is an important mechanism for the pro-angiogenic and anti-apoptotic function resulting from the knockdown of endothelial PDCD10 (12). The present study adds new evidence that NVP is able to reverse shPDCD10-induced activation of EphB4 thus its tumor-promoting effect via inhibiting EphB4 in GBM cells and in a mouse model of human GBM.

Loss of function mutations in PDCD10 causes defects in vasculogenesis and aberrant angiogenesis, and impairs vessel maturation/remodeling. Therefore, it is comprehensive that the knockdown of endothelial PDCD10 activates GBM cells and promotes tumor growth via stimulating multiple growth factors and angiogenic factors (34). However, the role of PDCD10 in tumor cells remains elusive and appears context dependent (18, 19, 35, 48–50). Barrier et al. (48) demonstrates that over-expression of PDCD10 is associated with poor prognosis of colorectal cancer patients. Ma et al. (50) points out that PDCD10 could promote prostate cancer cell proliferation and transformation by activating MST4 activity and may be involved in the Erk pathway. It is also demonstrated that as downstream target of miRNAs, PDCD10 is involved in the tumor phenotype (19) and chemo-resistance (18). More recently, Urfali-Mamatoglu et al. (49) reported that the dual function of PDCD10 in drug resistance. In the central nervous system, patients harboring heterozygous mutations in PDCD10 displayed a high risk for developing meningioma (15–17), suggesting a potential tumor suppressor-like function of PDCD10. The present study together with our recent work (35) report for the first time that GBM cells upon loss of PDCD10 and as a consequence activation of EphB4 exhibit a more aggressive behavior as shown by enhanced proliferation, migration, adhesion, and invasion *in vitro* as well as by the faster tumor progression *in vivo*. These findings are in agreement with so far identified major functions of EphB4 in GBM, i.e., promoting glioma cell migration and invasion (51). Moreover, we noted apparent functional tumor vessels in the shPDCD10-xenograft tumor. This phenomenon had been also observed in our previous study (35) and was confirmed in the present study. Given that the xenograft tumors developed after the implantation of GBM cells (U87) only without ECs, the formation of such microvessels might imply that loss of PDCD10 in GBM cells facilitates vascular mimicry by triggering a transformation of GBM cells to endothelial phenotype and stimulating tumor-angiogenesis. Importantly, inhibiting EphB4 activation significantly attenuated the tumor-angiogenesis *in vivo*. It is interesting to unveil if GBM cells transit to endothelial cells via PDCD10 and how PDCD10 regulates EphB4 expression in endothelial cells and in GBM cells in the further.

## Data Availability Statement

All datasets presented in this study are included in the article/Supplementary Material.

## Ethics Statement

The animal study was reviewed and approved by the Animal Center of the Medicine Faculty, University Hospital Essen, University of Duisburg-Essen.

## Author Contributions

US and YZ: conceptualization. XW, DS, SK, and PD: data curation. XW: formal analysis and writing – original draft. YZ: funding acquisition and project administration. XW and YW: investigation. XW, YW, KK, and YZ: methodology. XW and YZ: visualization. SK and YZ: writing – review & editing. All authors have read and agreed to the published version of the manuscript.

## Conflict of Interest

The authors declare that the research was conducted in the absence of any commercial or financial relationships that could be construed as a potential conflict of interest.

## Supplementary Material

Supplementary Figure 1 Correlation of EphB4 and PDCD10 expression in cohort of GBM (*n* = 156). A weak negative correlation was found between the two variables (*r* = −0.206; significance level = 0.01).

## References

[1] Alexander BM Cloughesy TF. Adult glioblastoma. J Clin Oncol. (2017) 35:2402–9. 10.1200/JCO.2017.73.0119 28640706

[2] Cloughesy TF Cavenee WK Mischel PS. Glioblastoma: from molecular pathology to targeted treatment. Annu Rev Pathol. (2014) 9:1–25. 10.1146/annurev-pathol-011110-130324 23937436

[3] Alifieris C Trafalis DT. Glioblastoma multiforme: pathogenesis and treatment. Pharmacol Ther. (2015) 152:63–82. 10.1016/j.pharmthera.2015.05.005 25944528

[4] Batchelor TT Reardon DA de Groot JF Wick W Weller M. Antiangiogenic therapy for glioblastoma: current status and future prospects. Clin Cancer Res. (2014) 20:5612–9. 10.1158/1078-0432.CCR-14-0834 25398844 PMC4234180

[5] De Bonis P Marziali G Vigo V Peraio S Pompucci A Anile C. Antiangiogenic therapy for high-grade gliomas: current concepts and limitations. Expert Rev Neurother. (2013) 13:1263–70. 10.1586/14737175.2013.856264 24175724

[6] Petit N Blecon A Denier C Tournier-Lasserve E. Patterns of expression of the three cerebral cavernous malformation (CCM) genes during embryonic and postnatal brain development. Gene Expr Patterns. (2006) 6:495–503. 10.1016/j.modgep.2005.11.001 16455310

[7] Chen L Tanriover G Yano H Friedlander R Louvi A Gunel M. Apoptotic functions of PDCD10/CCM3, the gene mutated in cerebral cavernous malformation 3. Stroke. (2009) 40:1474–81. 10.1161/STROKEAHA.108.527135 19246713 PMC2709460

[8] Zhu Y Wu Q Xu JF Miller D Sandalcioglu IE Zhang JM. Differential angiogenesis function of CCM2 and CCM3 in cerebral cavernous malformations. Neurosurg Focus. (2010) 29:E1. 10.3171/2010.5.FOCUS1090 20809750

[9] Bergametti F Denier C Labauge P Arnoult M Boetto S Clanet M. Mutations within the programmed cell death 10 gene cause cerebral cavernous malformations. Am J Hum Genet. (2005) 76:42–51. 10.1086/426952 15543491 PMC1196432

[10] Jenny Zhou H Qin L Zhang H Tang W Ji W He Y Endothelial exocytosis of angiopoietin-2 resulting from CCM3 deficiency contributes to cerebral cavernous malformation. Nat Med. (2016) 22:1033–42. 10.1038/nm.4169 27548575 PMC5014607

[11] You C Sandalcioglu IE Dammann P Felbor U Sure U Zhu Y. Loss of CCM3 impairs DLL4-Notch signalling: implication in endothelial angiogenesis and in inherited cerebral cavernous malformations. J Cell Mol Med. (2013) 17:407–18. 10.1111/jcmm.12022 23388056 PMC3823022

[12] You C Zhao K Dammann P Keyvani K Kreitschmann-Andermahr I Sure U. EphB4 forward signalling mediates angiogenesis caused by CCM3/PDCD10-ablation. J Cell Mol Med. (2017) 21:1848–58. 10.1111/jcmm.13105 28371279 PMC5571521

[13] Draheim KM Fisher OS Boggon TJ Calderwood DA. Cerebral cavernous malformation proteins at a glance. J Cell Sci. (2014) 127:701–7. 10.1242/jcs.138388 24481819 PMC3924200

[14] Zhou Z Tang AT Wong WY Bamezai S Goddard LM Shenkar R. Cerebral cavernous malformations arise from endothelial gain of MEKK3-KLF2/4 signalling. Nature. (2016) 532:122–6. 10.1038/nature17178 27027284 PMC4864035

[15] Riant F Bergametti F Fournier HD Chapon F Michalak-Provost S Cecillon M. CCM3 mutations are associated with early-onset cerebral hemorrhage and multiple meningiomas. Mol Syndromol. (2013) 4:165–72. 10.1159/000350042 23801932 PMC3666455

[16] Labauge P Fontaine B Neau JP Bergametti F Riant F Blecon A. Multiple dural lesions mimicking meningiomas in patients with CCM3/PDCD10 mutations. Neurology. (2009) 72:2044–6. 10.1212/WNL.0b013e3181a92b13 19506228

[17] Fauth C Rostasy K Rath M Gizewski E Lederer AG Sure U. Highly variable intrafamilial manifestations of a CCM3 mutation ranging from acute childhood cerebral haemorrhage to late-onset meningiomas. Clin Neurol Neurosurg. (2015) 128:41–3. 10.1016/j.clineuro.2014.10.023 25462093

[18] Zhang Y Hu X Miao X Zhu K Cui S Meng Q. MicroRNA-425-5p regulates chemoresistance in colorectal cancer cells via regulation of programmed cell death 10. J Cell Mol Med. (2016) 20:360–9. 10.1111/jcmm.12742 26647742 PMC4727563

[19] Fu X Zhang W Su Y Lu L Wang D Wang H. MicroRNA-103 suppresses tumor cell proliferation by targeting PDCD10 in prostate cancer. Prostate. (2016) 76:543–51. 10.1002/pros.23143 26771762

[20] Lambertz N El Hindy N Kreitschmann-Andermahr I Stein KP Dammann P Oezkan N. Downregulation of programmed cell death 10 is associated with tumor cell proliferation, hyperangiogenesis and peritumoral edema in human glioblastoma. BMC Cancer. (2015) 15:759. 10.1186/s12885-015-1709-8 26490252 PMC4618952

[21] Murai KK Pasquale EB. 'Eph'ective signaling: forward, reverse and crosstalk. J Cell Sci. (2003) 116:2823–32. 10.1242/jcs.00625 12808016

[22] Klein R. Eph/ephrin signaling in morphogenesis, neural development and plasticity. Curr Opin Cell Biol. (2004) 16:580–9. 10.1016/j.ceb.2004.07.002 15363810

[23] Mellitzer G Xu Q Wilkinson DG. Eph receptors and ephrins restrict cell intermingling and communication. Nature. (1999) 400:77–81. 10.1038/21907 10403252

[24] Cheng N Brantley DM Chen J. The ephrins and Eph receptors in angiogenesis. Cytokine Growth Factor Rev. (2002) 13:75–85. 10.1016/S1359-6101(01)00031-4 11750881

[25] Heroult M Schaffner F Augustin HG. Eph receptor and ephrin ligand-mediated interactions during angiogenesis and tumor progression. Exp Cell Res. (2006) 312:642–50. 10.1016/j.yexcr.2005.10.028 16330025

[26] Kumar SR Scehnet JS Ley EJ Singh J Krasnoperov V Liu R. Preferential induction of EphB4 over EphB2 and its implication in colorectal cancer progression. Cancer Res. (2009) 69:3736–45. 10.1158/0008-5472.CAN-08-3232 19366806

[27] Xia G Kumar SR Stein JP Singh J Krasnoperov V Zhu S. EphB4 receptor tyrosine kinase is expressed in bladder cancer and provides signals for cell survival. Oncogene. (2006) 25:769–80. 10.1038/sj.onc.1209108 16205642

[28] Brantley-Sieders DM Jiang A Sarma K Badu-Nkansah A Walter DL Shyr Y. Eph/ephrin profiling in human breast cancer reveals significant associations between expression level and clinical outcome. PLoS ONE. (2011) 6:e24426. 10.1371/journal.pone.0024426 21935409 PMC3174170

[29] Hasina R Mollberg N Kawada I Mutreja K Kanade G Yala S. Critical role for the receptor tyrosine kinase EPHB4 in esophageal cancers. Cancer Res. (2013) 73:184–94. 10.1158/0008-5472.CAN-12-0915 23100466

[30] Ferguson BD Liu R Rolle CE Tan YH Krasnoperov V Kanteti R. The EphB4 receptor tyrosine kinase promotes lung cancer growth: a potential novel therapeutic target. PLoS ONE. (2013) 8:e67668. 10.1371/journal.pone.0067668 23844053 PMC3699624

[31] Liu R Ferguson BD Zhou Y Naga K Salgia R Gill PS. EphB4 as a therapeutic target in mesothelioma. BMC Cancer. (2013) 13:269. 10.1186/1471-2407-13-269 23721559 PMC3671960

[32] Tu Y He S Fu J Li G Xu R Lu H. Expression of EphrinB2 and EphB4 in glioma tissues correlated to the progression of glioma and the prognosis of glioblastoma patients. Clin Transl Oncol. (2012) 14:214–20. 10.1007/s12094-012-0786-2 22374425

[33] Chen T Liu X Yi S Zhang J Ge J Liu Z. EphB4 is overexpressed in gliomas and promotes the growth of glioma cells. Tumour Biol. (2013) 34:379–85. 10.1007/s13277-012-0560-7 23138393

[34] Zhu Y Zhao K Prinz A Keyvani K Lambertz N Kreitschmann-Andermahr I. Loss of endothelial programmed cell death 10 activates glioblastoma cells and promotes tumor growth. Neuro Oncol. (2016) 18:538–48. 10.1093/neuonc/nov155 26254477 PMC4799675

[35] Nickel AC Wan XY Saban DV Weng YL Zhang S Keyvani K. Loss of programmed cell death 10 activates tumor cells and leads to temozolomide-resistance in glioblastoma. J Neuro Oncol. (2019) 141:31–41. 10.1007/s11060-018-03017-7 30392087

[36] Gholipour N Ohradanova-Repic A Ahangari G. A novel report of MiR-4301 induces cell apoptosis by negatively regulating DRD2 expression in human breast cancer cells. J Cell Biochem. (2018) 119:6408–17. 10.1002/jcb.26577 29236292

[37] Lodola A Giorgio C Incerti M Zanotti I Tognolini M. Targeting Eph/ephrin system in cancer therapy. Eur J Med Chem. (2017) 142:152–62. 10.1016/j.ejmech.2017.07.029 28780190

[38] Groppa E Brkic S Uccelli A Wirth G Korpisalo-Pirinen P Filippova M. EphrinB2/EphB4 signaling regulates non-sprouting angiogenesis by VEGF. EMBO Rep. (2018) 19:e45054. 10.15252/embr.201745054 29643120 PMC5934775

[39] Uhl C Markel M Broggini T Nieminen M Kremenetskaia I Vajkoczy P. EphB4 mediates resistance to antiangiogenic therapy in experimental glioma. Angiogenesis. (2018) 21:873–81. 10.1007/s10456-018-9633-6 29987450 PMC6208883

[40] Day BW Stringer BW Boyd AW. Eph receptors as therapeutic targets in glioblastoma. Br J Cancer. (2014) 111:1255–61. 10.1038/bjc.2014.73 25144626 PMC4183860

[41] Salgia R Kulkarni P Gill PS. EphB4: a promising target for upper aerodigestive malignancies. Biochim Biophys Acta Rev Cancer. (2018) 1869:128–37. 10.1016/j.bbcan.2018.01.003 29369779 PMC5955724

[42] Scehnet JS Ley EJ Krasnoperov V Liu R Manchanda PK Sjoberg E. The role of Ephs, ephrins, and growth factors in kaposi sarcoma and implications of ephrinb2 blockade. Blood. (2009) 113:254–63. 10.1182/blood-2008-02-140020 18836096 PMC2614637

[43] Djokovic D Trindade A Gigante J Badenes M Silva L Liu R. Combination of Dll4/Notch and Ephrin-B2/EphB4 targeted therapy is highly effective in disrupting tumor angiogenesis. BMC Cancer. (2010) 10:641. 10.1186/1471-2407-10-641 21092311 PMC3001720

[44] Krasnoperov V Kumar SR Ley E Li X Scehnet J Liu R. Novel EphB4 monoclonal antibodies modulate angiogenesis and inhibit tumor growth. Am J Pathol. (2010) 176:2029–38. 10.2353/ajpath.2010.090755 20133814 PMC2843490

[45] Martiny-Baron G Holzer P Billy E Schnell C Brueggen J Ferretti M. The small molecule specific EphB4 kinase inhibitor NVP-BHG712 inhibits VEGF driven angiogenesis. Angiogenesis. (2010) 13:259–67. 10.1007/s10456-010-9183-z 20803239 PMC2941628

[46] Becerikli M Merwart B Lam MC Suppelna P Rittig A Mirmohammedsadegh A. EPHB4 tyrosine-kinase receptor expression and biological significance in soft tissue sarcoma. Int J Cancer. (2015) 136:1781–91. 10.1002/ijc.29244 25274141

[47] Yang X Yang Y Tang S Tang H Yang G Xu Q. EphB4 inhibitor overcome the acquired resistance to cisplatin in melanomas xenograft model. J Pharmacol Sci. (2015) 129:65–71. 10.1016/j.jphs.2015.08.009 26390965

[48] Barrier A Lemoine A Boelle PY Tse C Brault D Chiappini F Colon cancer prognosis prediction by gene expression profiling. Oncogene. (2005) 24:6155–64. 10.1038/sj.onc.1208984 16091735

[49] Urfali-Mamatoglu C Kazan HH Gunduz U. Dual function of programmed cell death 10 (PDCD10) in drug resistance. Biomed Pharmacother. (2018) 101:129–36. 10.1016/j.biopha.2018.02.020 29482058

[50] Ma X Zhao H Shan J Long F Chen Y Chen Y. PDCD10 interacts with Ste20-related kinase MST4 to promote cell growth and transformation via modulation of the ERK pathway. Mol Biol Cell. (2007) 18:1965–78. 10.1091/mbc.e06-07-0608 17360971 PMC1877091

[51] Nakada M Anderson EM Demuth T Nakada S Reavie LB Drake KL. The phosphorylation of ephrin-B2 ligand promotes glioma cell migration and invasion. Int J Cancer. (2010) 126:1155–65. 10.1002/ijc.24849 19728339 PMC2801764

