# Supplementary figures and images for: PDCD10-Deficiency Promotes Malignant Behaviors and Tumor Growth via Triggering EphB4 Kinase Activity in Glioblastoma
Source: Front Oncol. 2020 Aug 7;10:1377. doi: 10.3389/fonc.2020.01377 (PMC7427606; doi:10.3389/fonc.2020.01377)

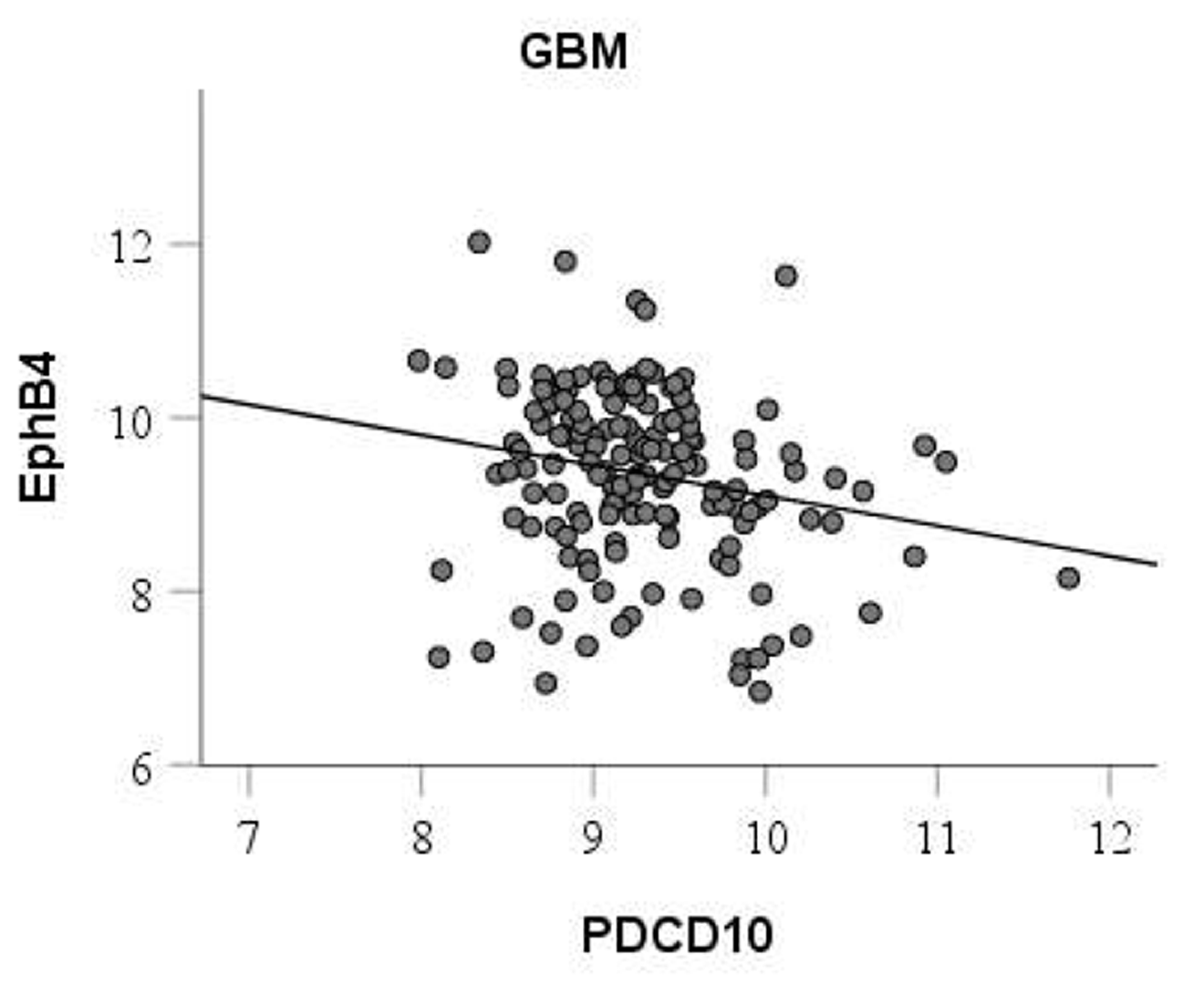

Supplement: Supplementary Figure 1 — Correlation of EphB4 and PDCD10 expression in cohort of GBM (n = 156). A weak negative correlation was found between the two variables (r = −0.206; significance level = 0.01). [file Image_1.TIF]
